# Emerging Genomic Tools for Legume Breeding: Current Status and Future Prospects

**DOI:** 10.3389/fpls.2016.00455

**Published:** 2016-05-02

**Authors:** Manish K. Pandey, Manish Roorkiwal, Vikas K. Singh, Abirami Ramalingam, Himabindu Kudapa, Mahendar Thudi, Anu Chitikineni, Abhishek Rathore, Rajeev K. Varshney

**Affiliations:** ^1^International Crops Research Institute for the Semi-Arid TropicsHyderabad, India; ^2^The University of Western AustraliaCrawley, WA, Australia

**Keywords:** trait mapping, gene discovery, genomics-assisted breeding, high-throughput genotyping, next-generation sequencing

## Abstract

Legumes play a vital role in ensuring global nutritional food security and improving soil quality through nitrogen fixation. Accelerated higher genetic gains is required to meet the demand of ever increasing global population. In recent years, speedy developments have been witnessed in legume genomics due to advancements in next-generation sequencing (NGS) and high-throughput genotyping technologies. Reference genome sequences for many legume crops have been reported in the last 5 years. The availability of the draft genome sequences and re-sequencing of elite genotypes for several important legume crops have made it possible to identify structural variations at large scale. Availability of large-scale genomic resources and low-cost and high-throughput genotyping technologies are enhancing the efficiency and resolution of genetic mapping and marker-trait association studies. Most importantly, deployment of molecular breeding approaches has resulted in development of improved lines in some legume crops such as chickpea and groundnut. In order to support genomics-driven crop improvement at a fast pace, the deployment of breeder-friendly genomics and decision support tools seems appear to be critical in breeding programs in developing countries. This review provides an overview of emerging genomics and informatics tools/approaches that will be the key driving force for accelerating genomics-assisted breeding and ultimately ensuring nutritional and food security in developing countries.

## Introduction

The demand-supply gap of food grain is continuously increasing due to the ever-growing global population which is likely to expand from 7.2 billion to 9.6 billion by 2050 and 10.9 billion by 2100 (Gerland et al., 2014). This burgeoning problem is becoming serious as the current yield increase trends may not be sufficient in dealing with the growing demand (Ray et al., 2013; Fedoroff, 2015). The speedy depletion of natural resources and climate change have badly affected the ongoing efforts to achieve higher productivity. In order to ensure hunger-free society with nutritious food, it is a challenge before the policy makers, farming community, and agriculture scientists to ensure nutritional food security by producing 60% higher food grain by 2050. Among the food grains, the grain legumes are the key sources of protein, minerals, vitamins, iron, zinc, calcium, and magnesium, as well as omega-3 fatty acids. The importance of these legumes is higher where a large section of the society depends on vegetarian food such as in India. The oilseed legume crops such as groundnut (*Arachis hypogaea*) and soybean (*Glycine max*) play important role in the production of cooking oil and other confectionaries preparations to the consumers. The unique ability to fix atmospheric nitrogen by the legume crops play a crucial role in sustaining the farming system by making available the residual nitrogen to the non-legume crops. Legumes also serve as an excellent source of high quality and nutritious feed to livestock leading to ∼20% increase in animal productivity (Tarawali and Ogunbile, 1995). The human civilization has a long association with legume cultivation, i.e., since 6000 years which has contributed significantly toward economical, nutritional, and improving the livelihood of subsistence farmers across the world.

It is well known that proteins are essential macronutrient for growth as well as maintenance of human body and a minimum protein intake of 0.8, 1.5, and 1.0 g protein/kg body weight/day is recommended for adults, children, and adolescents, respectively (Kafatos and Hatzis, 2008). The major protein sources include meat, fish, eggs, poultry, dairy products, legumes, nuts, and grains. Among legumes, the highest proportion of protein content is in soybean (33–45%) followed by common bean (*Phaseolus vulgaris*; 21–39%), winged bean (*Psophocarpus tetragonolobus*; 30–37%), cowpea (*Vigna unguiculata*; 21–35%), groundnut (24–34%), mung bean (*Vigna radiata*), pea (*Pisum sativum*; 21–33%), moth bean (*Vigna aconitifolia*), urd bean (*Vigna mungo*; 21–31%), lentil (*Lens culinaris*; 20–31%), grass pea (*Lathyrus sativus*; 23–30%), chickpea (*Cicer arietinum*; 15–30%), horse gram (*Macrotyloma uniflorum*), pigeonpea (*Cajanus cajan*; 19–29%), and rice bean (*Vigna umbellata*; 18–27%; Salunkhe et al., 1985). The productivity of these legumes could not be enhanced enough to meet the requirement, owning to several biotic and abiotic factors. In addition, the decreasing land and water resources together with climatic fluctuations will make situation worse, leading to protein unavailability to the human population in future. Due to ever increasing global population, especially in Asia where consumption of legumes is more, it is very important to increase the productivity of these legumes in order to meet the protein requirements for the future population.

The grain legumes cover approximately 71.8 million hectares with 50% area in Asia and 25% area in Africa. Although the productivity of legumes is 50% less than cereals, legumes fetch higher returns in the global market^1^. Although conventional breeding approaches have been successful to address the issue of low productivity in some legumes, this is not happening at the desired success rate. Therefore, it is very essential to intensify the legume genetic enhancement programs using advanced breeding approaches wherein the potential of genomics needs to be exploited for accelerated development of improved cultivars possessing high yield, genetic resilience against stresses, and enhanced nutritional quality. The next-generation sequencing (NGS) and genotyping technologies need to be used for precise marker-trait association, gene discovery, functional marker development, and their deployment in routine breeding programs. The detailed information on the availability of genetic and genomic resources in important legumes has been extensively reviewed in many articles (see Varshney et al., 2013c; Bohra et al., 2014; Varshney, 2016). In this review article, we have focused on the availability and deployment of modern and advanced genetic and genomic tools for conducting high resolution trait mapping and molecular breeding in three important legumes largely cultivated in semi-arid tropic (SAT) regions of the world, i.e., chickpea, pigeonpea, and groundnut. Further details on other emerging biological approaches for speedy identification of candidate genes and delivery of improved cultivars have also been provided, though some of these approaches are yet to be exploited in improving productivity, quality, and nutritional richness in these three important legumes.

## Next – Generation Genotyping and Sequencing Technologies

Several new genotyping platforms that leverage NGS technologies to discover and simultaneously genotype single nucleotide polymorphisms (SNPs) are currently available. As a result, the sequence-based genotyping (SbG) methods are becoming popular and method of choice for understanding the genetics of complex traits both in plants and animals. So far the SbG technologies have been deployed extensively in genetic mapping, purity testing, establishing marker-trait associations, marker-assisted selection (MAS), and genomic selection (GS) for crop improvement (see Varshney et al., 2014b). These technologies can be broadly classified as (i) amplicon based targeted sequencing, (ii) reduced representation based sequencing, and (iii) hybridization based approaches. Above mentioned technologies are discussed in details at http://www.illumina.com/Documents/products/appspotlights/app_spotlight_ngg_ag.pdf. The choice of platform for genotyping depends on several factors like the scale of genotyping project, genome size, and availability of funds. For instance, MAS, requires flexible, low-cost systems like LGC’s automated systems for running KASP^TM^ markers for genotyping smaller numbers of SNPs across large breeding populations. On the other hand, a wide range of options are available for custom genotyping of various number of samples × SNPs from Illumina and Affymetrix Technologies, Fluidigm’s Dynamic Arrays^TM^, Douglas Scientific’s Array Tape^TM^ for whole genome scanning, constructing high density genetic maps, and genome-wide association studies (GWAS; see Thompson, 2014). In this review, details have been provided on examples of deployment of SbG technologies for legume research and breeding, with some insights into future technologies that will accelerate legume breeding efforts.

Amplicon based targeted sequencing is amenable for addressing questions in population genetics and systematics that rely on sequence specific genes of known function or diversity levels (Naj et al., 2011). However, lack of inexpensive and fast approaches that allow rapid library preparation using a standard PCR product of specific gene for thousands of samples and sometimes for 100s of loci that are essential for establishing phylogenetic relationships necessitate the development of targeted amplicon sequencing approach (Bybee et al., 2011). Several approaches that improve the throughput by barcoding targeted amplicon have been extensively discussed in Mamanova et al. (2011). In fact, amplicon based sequencing targets only limited number of loci and is not useful for fine mapping of complex traits where genome-wide SNPs are essential. Nevertheless, genome-wide SNP calling often hindered by the complexity at genome level in case of some crops such as maize, wheat, groundnut, and soybean. Hence, reduced-representation based sequencing approaches that include reduced-representation libraries (RRLs) or complexity reduction of polymorphic sequences (CRoPS), restriction-site-associated DNA sequencing (RAD-seq) and low coverage genotyping were developed and are being deployed. Further, van Orsouw et al. (2007) developed an approach known as CRoPS^TM^ which is based on restriction digestion of DNA using methylation sensitive restriction enzyme that reduces the complexity of two or more genetically diverse samples are prepared by AFLP. High conversion rate to genotyping assays makes it more attractive for medium to large scale genotyping particularly for SNPs in low or single copy genome sequences. While Miller et al. (2007) proposed the reduced representation library in which the size reduction was carried out using restriction enzymes, this technique was adapted to include barcoding and multiplexing with Illumina sequencing technologies (Baird et al., 2008). In addition, Double Digest Restriction Associated DNA (ddRAD) sequencing developed by Peterson et al. (2012) exclusively uses size selection to recover an appropriate number of regions, which are distributed randomly throughout the genome and maximizes the ability of multiplexing of several 100s of samples. Nevertheless, the genotyping-by-sequencing (GBS) approach which has been extensively utilized in crop improvement programs (Kim et al., 2016), is more powerful compared to the RAD sequencing approach because the SNP discovery and genotyping can be done at the same time (Poland et al., 2012). Further, compared to the RAD method, GBS is substantially less complicated in terms of generation of restriction fragments with appropriate adapters, fewer DNA purification steps, and no fragments selection steps. Recently, a novel GBS approach called skim-based GBS (skimGBS) that uses low-coverage whole genome sequencing (WGS) for high-resolution genotyping has been developed (Bayer et al., 2015). Using this approach, genome-wide recombination maps can be developed, and the frequency of crossover as well as gene conversion events can be assessed and compared. The SkimGBS, is a two-stage method that requires a reference genome sequence, genomic reads from parental individuals, and individuals of the population. The parental reads are initially mapped to the reference genome and SNPs are called using SGSautoSNP (Lorenc et al., 2012). Subsequently progeny reads are mapped to the same reference and comparison with the parental SNP file enables the calling of SNPs between parental genotypes and progeny.

In recent years, exome sequencing technique is gaining importance as this enables discovery of many low-frequency and rare coding variants that need to be examined systematically for association with complex traits in both plants and animals (Kiezun et al., 2012). Various exome sequencing platforms and their application for crop improvement as well as health are discussed in detail (see War et al., 2015). Affymetrix and Illumina exome arrays can code the variations like SNPs and InDels and can offer custom content. Nevertheless, Affymetrix exome arrays have capacity for 100,000 additional markers compared to Illumina exome arrays. In addition to the exome arrays, high density SNP arrays have shown great promise as an efficient alternative to GBS approach often constrained by missing data and complex bioinformatics analysis (Unterseer et al., 2014). The higher efficiency in providing genotyping data for the most of the loci across the samples have made this approach most suitable for use in GS breeding, wherein, the consistent data is required not only in training population (TP) but also for all the consequent breeding lines subjected for estimating genomic estimated breeding values (GEBVs). The high density SNP arrays have been successfully developed and deployed in rice (*Oryza sativa*; 50 K SNPs; Singh N. et al., 2015) and maize (*Zea mays*; 616 K SNPs; Unterseer et al., 2014) and will be available in several other important crop plants in coming days. With an objective to exploit the potential of such SNP arrays, the Affymetrix arrays with 60 K SNPs for three legume crops viz., chickpea, pigeonpea, and groundnut were developed by ICRISAT recently (see Varshney, 2016). The availability of these arrays will provide high-throughput, cost-effective, reproducible, and informative SNP genotyping data to facilitate high resolution trait mapping and molecular breeding.

## Sequencing and Re-Sequencing Efforts in Legumes

Decoding of the plant genome sequence provides an opportunity to dissect and understand the mechanism or genetic basis for functional characterization of genes. Considering the importance of genome sequencing for crop improvement, several plant genomes have been decoded (Michael and Jackson, 2013). *Arabidopsis* (*Arabidopsis thaliana)* was the first plant species for which genome sequencing was completed (The Arabidopsis Genome Initiative, 2000) followed by three efforts to sequence *indica* and *japonica* rice (Goff et al., 2002; Yu et al., 2002; International Rice Genome Sequencing Project [IRGSP], 2005) using Sanger sequencing technology. NGS technology based WGS approach could reduce the time and cost of genome sequencing drastically as compared to Sanger sequencing (Schatz et al., 2002). Adoption of NGS technologies made it possible to sequence genome in much less time and cost, which encouraged researchers to even decode the complex genome sequences. By July 2013, >50 plant genomes were sequenced (Michael and Jackson, 2013) and more than 100 have been added since then. It is important to note that majority of these genomes were sequenced using either only NGS technologies or in combination of NGS and Sanger sequencing technologies.

Until recently, legumes namely chickpea, pigeonpea, and groundnut were considered orphan crops as not much genomic resources were available (Varshney et al., 2012b). With the advent of NGS technologies, efforts were made to develop genomic resources for these crops (Varshney et al., 2013c). Among these legumes, genome sequence of the pigeonpea was first to be completed in 2012 (Varshney et al., 2012a). Illumina NGS technology along with Sanger based bacterial artificial chromosome (BAC) end sequences was used for assembling the pigeonpea genome. ICPL 87119 (also known as ‘Asha,’ an inbred line and a widely cultivated medium duration pigeonpea genotype) was sequenced using Illumina sequencing technology to generate 237.2 Gb of paired-end (PE) reads. Filtered 130.7 Gb high quality Illumina sequencing data and Sanger sequencing data for >88 K BACs were used to assemble 605.78 Mb representing 72.7% of the pigeonpea genome. Gene annotation using combination of *de novo* gene prediction and homology-based methods led to identification of ∼48 K genes, though it seems to be on a higher side, in pigeonpea genome (Varshney et al., 2012a). All these genes were functionally annotated using combination of a range of approaches, which resulted in assigning tentative gene function to more than 95% of genes and less than 4% genes could not be functionally annotated. In an another effort to sequence the pigeonpea genome, long sequence read of 454 GS-FLX pyrosequencing technology were used for assembling ∼548 Mb of pigeonpea genome (Singh N.K. et al., 2012).

Next-generation sequencing approach was also used for sequencing the chickpea genome of CDC Frontier (a *kabuli* chickpea variety) by International Chickpea Genome Sequencing Consortium (ICGSC). Around 153 Gb sequence data were generated using Illumina sequencing technology of which 87.65 Gb of high-quality sequence data were assembled into 544.73 Mb of genomic sequence scaffolds representing 74% of chickpea genome (Varshney et al., 2013d). Using a combination of approaches, more than 28 K non-redundant gene models were predicted across the chickpea genome of which around 25 K (89.73%) could be functionally annotated. Along with the draft chickpea genome, sequence data were generated for 90 chickpea accessions using whole genome re-sequencing (WGRS) and RAD-sequencing approaches (Varshney et al., 2013d). In another effort to sequence the chickpea genome, ICC 4958 *desi* chickpea genotype was targeted for generating a draft genome assembly using NGS platforms along with BAC end sequences and a genetic map. A total of 13.35 Gb high quality sequencing data from 454/Roche GS FLX Titanium platform and 43.7 Gb of quality-filtered PE Illumina sequence data on ICC 4958 along with BAC end sequences were used to assemble ∼520 Mb of chickpea genome (Jain et al., 2013). Very recently, an improved version of *desi* chickpea cultivar ICC 4958 with 2.7-fold increase in the length of pseudomolecules was reported (Parween et al., 2015). This improved assembly could reduce the gaps in the existing genome assembly and predicted the presence of more than 30 K protein-coding genes. In a similar effort, Ruperao et al. (2014) used chromosomal sequencing approach to validate the available *desi* and *kabuli* chickpea genome assemblies. Isolation and NGS based sequencing of individual chromosomes helped in validating the genome assemblies at chromosome level, which identified small misassembled regions in *kabuli* and large misassembled region in *desi* draft genomes (Ruperao et al., 2014).

Very recently, the International Peanut Genome Initiative (IPGI) successfully sequenced the genomes of diploid groundnut progenitors. The progenitors representing A-genome (*Arachis duranensis*, accession V14167) and B-genome (*Arachis ipaensis*, accession K30076) together represent the tetraploid genome of cultivated groundnut (*Arachis hypogaea*). In this context, a total of 216 Gb WGS data at 154.4X coverage for accession V14167 while 168.8 Gb data at 120.6X coverage for accession K30076 were generated (Bertioli et al., 2016). In addition, 155.5 Mb transcriptomic sequence data for accession V14167 and 175.6 Mb data for accession K30076 were generated from different plant tissues and growth stages. The above mentioned sequences were assembled into 10 pseudomolecules for each genome. The B-genome (1.37 Gb) had larger genome size than A-genome (1.2 Gb). The availability of the genome sequence will provide access to 97% of groundnut genes in their genomic context to the global groundnut research community leading to development of better understanding about the complex groundnut genome and accelerated development of more productive, climate, and stress resilient groundnut varieties.

The draft genome sequence serves as the foundation for deploying genomics in crop improvement for accelerating the rate of genetic gains by identifying the genes responsible for economically important traits. The draft genome sequence also helps to understand the genome architecture and unravel the basic mechanism involve in the stress responses. The availability of draft genome sequence has enabled the undertaking of large scale genome re-sequencing projects for identification of genetic variations, single base mutations, insertions, and deletions. Following the completion of draft reference genome, cataloging the sequence variations by comparing the genome sequence at individual and population level helps in understanding the mechanism of plant’s response to different stresses. Large scale germplasm resources available in genebanks world-wide provide opportunity to the global plant science community to mine superior alleles (McCouch et al., 2013). ICRISAT’s genebank has about 50,000 accessions of cultivated species and wild relatives of chickpea, pigeonpea, and groundnut from 133 countries (Gowda et al., 2013). In order to identify new sources of genetic variation and allelic variants of candidate gene(s) associated with beneficial traits by exploring the huge genetic diversity available in the genebank, ICRISAT has initiated the efforts to re-sequence the germplasm for identification of novel alleles. In the case of pigeonpea, 292 *Cajanus* accessions from reference set were re-sequenced using WGRS approach and were used to identify 13.8 million sequence variations (SNPs and InDels) with average of 41.1 variations per Kb (Saxena et al., 2015). Detailed analysis of re-sequencing data provided genetic variation patterns across the pigeonpea genome along with identification of genomic regions that are expected to play an important role during domestication and selection. Besides improved understanding of the genome, such genetic variations together with phenotypic data are also being used for undertaking marker-trait association analysis for identification of markers associated with trait of interest. In addition, 104 pigeonpea hybrid parental lines were also re-sequenced using WGRS approach. The hybrid parental lines includes, CMS lines, maintainer and restorer lines. The generated genotyping datasets along with phenotyping of parental lines and there of derived hybrids will be utilized for construction of heterotic pool in pigeonpea.

In the case of chickpea, WGRS approach was used for resequencing >400 chickpea genotypes and were analyzed to identify ∼4.7 million SNPs, >500,000 Indels and CNVs. Resequencing data on >100 elite chickpea varieties were used for developing first generation Hapmap of chickpea. Re-sequencing data on 300 lines form chickpea reference set along with available phenotyping data is being used for identification of markers associated with trait of interest. Considering the utility/application of WGRS in the crop improvement program, very recently ICRISAT had launched “The 3000 Chickpea Genome Sequencing Initiative” where 3000 lines from the global composite collection of chickpea from genebank of ICRISAT and ICARDA are being re-sequenced for identification of novel alleles (see Varshney, 2016).

## Advanced/Multi-Parent Mapping Populations for High Resolution Mapping

**FIGURE 1:**
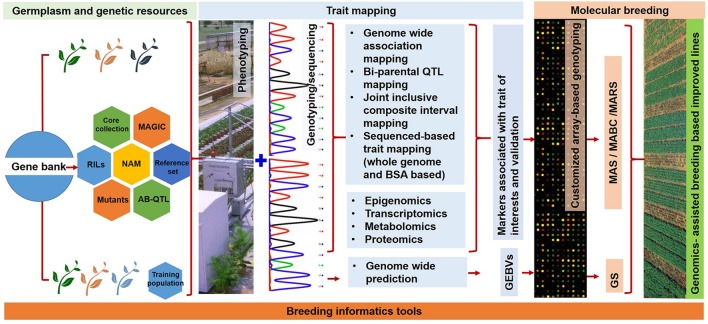
**A flowchart showing different stages of trait discovery and trait deployment using NGS approaches for achieving higher genetic gains and accelerated development of improved genotypes.** The different germplasm sets (such as core collection, reference set, and mutants) and genetic populations (RILs, NAM, MAGIC, and AB-QTL) provide genetic variation for agronomically important traits. These populations are subjected to next generation sequencing and high-throughput genotyping, and high quality phenotyping leading to the discovery of candidate genes and trait specific diagnostic markers. Such markers are prerequisite for deploying MAS, MABC, and MARS in crops. Different “omics” approaches can play an important role in achieving better understanding and greater insights for the target traits. In addition, the deployment of genomic selection breeding will help in achieving higher genetic gains in less time.

For achieving accelerated genetic gains, a number and combination of genomics-assisted breeding (GAB) approaches need to be used. Marker-assisted backcrossing (MABC) for improvement of single or multiple traits (gene pyramiding) is the most successful GAB approach which has helped in the development of several improved varieties and lines in many crops. However in order to deploy GAB, the identification of genomic regions either through linkage mapping or association mapping is mandatory for the traits of interest. There are three trait mapping approaches using forward genetics namely (1) linkage mapping/quantitative trait locus (QTL) mapping, (2) association mapping/linkage disequilibrium (LD) mapping, and (3) joint linkage-association mapping (JLAM). Majority of the linkage mapping studies used either F_2_, recombinant inbred lines (RILs), backcross inbred lines (BILs), near isogenic lines (NILs), advanced intercrossed lines (AILs), or double haploid populations in crop plants. On the other hand, germplasm sets were used for association mapping. All the above mentioned mapping populations had a major disadvantage that only few traits can be mapped. Realizing the long time spent in developing these populations, the recent trend has shifted in development and deployment of multi-parent mapping populations. These populations include nested association mapping (NAM) and multi-parent advanced generation inter-cross (MAGIC) populations (Varshney and Dubey, 2009) (**Figure 1**). Gene discovery activities in the past were limited by the extent of availability of genomic data in a crop but with NGS technology there is a need to redesign the way to understand the genetics of traits of interest. These populations have advantages of both bi-parental (high power of QTL detection) and association mapping (high resolution; Gupta et al., 2014). Therefore, these two populations achieve higher level of polymorphism, high resolution genetic mapping and QTL identification, and handling several traits in one go.

### Advanced Intercrossed Line (AIL)

Development of large bi-parental population to achieve higher recombination events is not a cost-effective approach due to substantial cost involved in developing, phenotyping, and genotyping. In order to achieve higher resolution within a small population, it is important to increase the recombination events for the target loci. In this context, AIL population is very promising approach where in the two variant lines selected in F_2_ generation are randomly and sequentially intercrossed to several generations (Darvasi and Soller, 1995). Such crossing efforts break linkage even between very closely linked loci due to several recombination events. Development and deployment of such populations may provide more precise location and estimates of genomic/quantitative trait loci, and therefore, a good genetic resource for fine mapping. Such mapping populations were largely deployed for fine mapping in several plant species (Huang X. et al., 2010). However such populations have not been deployed frequently in these legume crops.

### Nested Association Mapping (NAM)

This approach takes benefits of both mapping approaches, i.e., linkage and association mapping and has shown ability to identify and resolve functional markers for complex traits. It is important to note that the success depends upon frequency of functional marker alleles, magnitudes of their genetic effects, disequilibrium among functional and non-functional markers, statistical analysis methods, and mating design (Guo et al., 2010). Technically, the NAM population makes use of both primitive and recent recombination events to take advantage of low marker density requirements, allele richness, high mapping resolution, and high statistical power. Since this approach involves several parental genotypes which led to generation of lines from nested mating designs, and thereby more chance of resolving the functional markers as compared to the bi-parental populations.

The NAM populations are currently being developed at ICRISAT (see Varshney, 2016). In the case of chickpea, with an objective to generate new breeding material with enhanced diversity for high resolution mapping of target traits, ICRISAT along with its partner is developing NAM population having ICC 4958 as common female parent. In total 14 different crosses were initiated and generated F_1_s. These F_1_s are being advanced to generate minimum of 200 lines from each cross. In pigeonpea, for mapping different traits (FW, SMD, yield and yield related traits and seed protein content), NAM population is being developed. In this regard 10 different F_1_ combinations were generated by crossing Asha variety (ICPL 87119) with 10 different elite pigeonpea lines. As a result a total of 10 NAM-F_2:3_ populations were developed. These developed populations are currently being genotyped through GBS based approach and phenotyped for the target traits for identification of marker-trait associations. Similarly, in the case of groundnut, two NAM populations, i.e., one each for Spanish type (ICGV 91114 and 22 testers) and Virginia type (ICGS 76 and 21 testers) are being developed at ICRISAT.

### Multi-parent Advanced Generation Inter-Cross (MAGIC)

The MAGIC population is generated from multiple parents (4–8) of diverse origins including alien backgrounds possessing desired features to bring multiple favorable alleles in multiple combinations in the population. This population allows both coarse and fine mapping and the complex architecture of many traits which are associated with crop yield and quality can be deduced using epistatic interactions (Cavanagh et al., 2008). MAGIC populations, in future, will serve as important resources for the discovery, isolation, and transfer of essential genes to facilitate crop improvement.

The MAGIC populations are currently being developed at ICRISAT (see Varshney, 2016). In the case of chickpea a MAGIC population with around 1200 lines were developed using eight parents including cultivars and elite breeding lines (ICC 4958, ICCV 10, JAKI 9218, JG 11, JG 130, JG 16, ICCV 97105, and ICCV 00108) from India and Africa. These eight diverse parents were crossed in 28 two-way, 14 four-way, and 7 eight-way crosses for accumulation of recombination events to allow genome reshuﬄe to bring greater genetic diversity (see Varshney, 2016). In order to access the allele diversity in the MAGIC population, the population have been re-sequenced using WGRS approach and generated a total of 4.67 Tb clean sequence data. Alignment of re-sequence data to the reference genome led to identification of 1 million SNP variants. These SNPs are being used for further understanding genome diversity and haplotype analysis.

In pigeonpea, MAGIC population is also being developed to enhance the genetic base and to identify the marker trait associations. A total of eight diverse founder parents (ICP 5529, HPL 24, ICP 7035, ICP 8863, ICP 4486, ICP 11605, ICP 7426, and ICP 14209) were selected for the development of MAGIC population. Half di-allele crossing approach (28, two-way F_1_s) followed by funnel based mating design (14 four-way and 7 eight-way F1s) was utilized for the development of MAGIC lines. Currently, 7 eight-way F_1_s are being selfed in the controlled conditions for the development of high resolution MAGIC lines. In the case of groundnut, three MAGIC populations are under development targeting different trait combinations. The first MAGIC population (ICGV 88145, ICGV 00308, ICGV 91114, ICGV 06040, ICGV 00440, ICGV 05155, GPBD 4, and 55-437) targets different traits such as fresh seed dormancy, oil content, seed mass, kernel Fe and Zn content, aflatoxin tolerance, stem rot tolerance, and PBND tolerance. The second MAGIC population targets the different components of aspergillus resistance and aflatoxin contamination while the third one targets different component traits of drought tolerance. The genotypes for aflatoxin resistance include 55-437, ICG 51, ICGV 12014, U4-7-5, VRR245, ICGV 88145, ICGV 89104, and ICGV 97278 while for drought tolerance include ICGV 02022, ICG 7190, ICGV 97183, ICG 3053, ICG 14482, ICG 11515, TAG 24, and ICGV 02266.

## Sequencing-Based Trait Mapping in Legumes

Advances in NGS technologies and a steeper drop down in the cost of sequencing provides a significant opportunity for trait mapping at sequence level and selection of plants at nucleotide levels. Traditional approaches of trait mapping were time-consuming and costly in comparison to NGS-based approaches. Recently, a large number of sequencing-based approaches have been proposed and used for trait mapping, which can be mainly classified in two groups (i) trait mapping through sequencing of complete populations and (ii) trait mapping through sequencing of pooled samples (**Figure 1**). The brief description of the specific approaches and current status in different legumes are given below.

### Trait Mapping through Sequencing of Complete Populations

The GBS and WGRS of mapping populations provide large scale genome-wide SNPs for conducting high resolution trait mapping.

The GBS (Elshire et al., 2011) has been found promising approach for rapid identification of large number of genome-wide SNPs for diversity assessment, trait mapping, GWAS, and GS in several crops (see He et al., 2014). GBS has gained popularity due to low genotyping cost for providing high-density genotyping data. In addition, it also provides the edge over other sequencing-based methods as this does not require the prior genome information. GBS has been extensively used for genetic diversity analysis (Fu et al., 2014), developing dense genetic maps (Kujur et al., 2015b; Li et al., 2015), refine the target genomic regions (Jaganathan et al., 2015), marker-trait associations (Romay et al., 2013) as well as deployment in GS breeding (Poland et al., 2012 in wheat, Crossa et al., 2013 in maize, and Huang et al., 2014 in oat). Applications of GBS in crop improvement have been extensively reviewed in many research papers (see Heschamps et al., 2012; Poland and Rife, 2012; He et al., 2014; Kim et al., 2016). For instance, GBS approach was used successfully for enriching the existing genetic map of chickpea developed with 241 SSR loci (Varshney et al., 2014c) to 1,007 loci including 828 SNPs identified using GBS along with earlier mapped SSRs (Jaganathan et al., 2015). Interestingly GBS could saturate the “*QTL-hotspot*” region identified by Varshney et al. (2014c) that harbors QTLs for drought tolerance component traits, by integrating 45 additional GBS-SNPs narrowing down the genomic region from 29 cM to 14 cM (Jaganathan et al., 2015). Additionally, GBS based approach was used for development of high-density linkage map in chickpea comprising of 1,336 SNPs (Deokar et al., 2014). In another study, one genetic map each for *desi* and *kabuli* type chickpea were reported with 3,625 SNPs and 2,177 SNPs, respectively. These high-density linkage maps were then used for identification of QTLs controlling seed weight in chickpea (Kujur et al., 2015b). GBS and Skim sequencing approaches also demonstrated their utility in improving the genome assemblies of both *desi* and *kabuli* chickpeas (Ruperao et al., 2014), which serve as a better reference for legume biology and comparative genomics. Complex admixed domestications patterns of chickpea were reported using high-throughput GBS approach (Kujur et al., 2015a). GBS approach is also being utilized for generating genotyping data on ∼20 mapping populations segregating for diverse targeted traits including fusarium wilt (FW) and sterility mosaic disease (SMD) resistance, seed protein content, yield and its associated traits in pigeonpea. Similarly, in groundnut the mapping population (TAG 24 × GPBD 4) is being genotyped using GBS approach for identification of candidate genomic regions for leaf rust, late leaf spot, and other agronomic important traits (see Varshney, 2016).

The WGRS of entire mapping population or diverse germplasm set is one of the promising approaches for many diverse studies; including identification of candidate genomic region/gene and GWAS (see Huang et al., 2013). The classical example of GWAS through WGRS was reported by Huang Y.-F. et al. (2010) in rice which identified marker trait associations for 14 agronomic traits after generating ∼3.6 million SNPs by sequencing 517 rice landraces map. Similarly, to dissect the genetic architecture of oil biosynthesis in maize kernels, a total of 368 maize inbred lines were sequenced to perform GWAS (Li et al., 2013). This study identified 26 loci associated with oil concentration explaining up to 83% phenotypic variation. In the context of legumes, WGRS for performing GWAS is currently being utilized in 292 lines of reference set and 104 hybrid parental lines to define heterotic pool in pigeonpea (see Varshney et al., 2015b). Similarly, in the case of chickpea ∼300 lines of reference set and 100 elite varieties have been sequenced for performing GWAS for economically important traits. In addition to this “The 3,000 Chickpea Genome Sequencing Initiative” has also been started to capture superior alleles for the targeted traits (see Varshney, 2016). In addition to GWAS, the first successful example of WGRS in segregation mapping population for identification of candidate gene(s) was reported in rice by Huang et al. (2009). Sequencing of RIL population with 150 individuals at lower coverage (∼0.02× coverage) resulted in identification of 1,493,461 SNPs with average density of 25 SNPs/Mb or 1 SNP every 40 kb. The detailed analysis lead identification of 100-kb region containing the rice “green revolution” gene (Huang et al., 2009). In another study for pinpointing genes for root-knot nematode (RKN) resistance in soybean, a total of 246 RILs were sequenced at an average of 0.19X depth and identified two candidate genes (*Glyma10g02150* and *Glyma10g02160*) associated with RKN resistance in soybean (Xu et al., 2013). In this context, examples of WGRS of mapping population in the ICRISAT-mandate legume crops are still not reported, however, in near future with low cost of sequencing, it will be feasible to generate WGRS data for entire mapping population in these legumes as well.

It is important to note that the sequencing of mapping populations/genotypes through GBS approach suffers from the limitation of missing regions of the genome whereas the WGRS overcomes the missing data challenge. On the other hand, the WGRS offers better data quality, however, it is also a costly process. To save the cost of sequencing, WGRS can be done at lower depth and in that scenario the approach is referred as skim sequencing (Golicz et al., 2015). Using this approach, Bayer et al. (2015) characterized the distribution of crossover and non-crossover recombination in rape mustard (*Brassica napus*) and chickpea using SkimGBS. GWAS based gene enrichment analysis of skim sequenced data of RIL population in chickpea was useful to split the earlier identified “*QTL-hotspot*” in two sub-regions viz. “*QTL-hotspot_a*” and “*QTL-hotspot_b*” of 139.22 and 153.36 Kb sizes, respectively (Kale et al., 2015). For targeting direct candidate genes from the segregating mapping populations and or diverse germplasm set, exome sequencing approach has been proposed. This is the most promising approach for sequencing all the protein-coding genes in a genome (known as the exome). This approach is also useful for those crops which are having higher genome size like groundnut, maize, barley (*Hordeum vulgare*) and wheat (*Triticum aestivum*; see Singh D. et al., 2012; War et al., 2015). Exome sequencing approach is currently being utilized to sequence 250 lines of groundnut for understanding the role of candidate genes for the targeted traits. Identification of non-synonymous SNPs substitution in the identified candidate genomic regions or through identification of putative nsSNPs through principal component analysis between a different set of genotypes is one of the promising approach. Recently, this approach has been used for identification of nsSNPs of the target candidate genes for sheath blight resistance in rice (Silva et al., 2012) and more complex trait like the drought tolerance in maize (Xu et al., 2014). Based on the advantages of this approach, it has also been utilized for the identification of candidate genes for FW and SMD resistance in pigeonpea (Singh V.K. et al., 2015).

### Trait Mapping through Pooled Sequencing

There are currently five trait mapping approaches wherein the sequencing of complete population is not required and the analysis is done on the sequences generated on pooled samples. These include QTL-Seq, MutMap, Seq-BSA, Indel-Seq, and BSR-Seq.

The “QTL-Seq” is the first, and most promising approach which has been successfully applied in crop plants with higher genome size. It has been utilized in the localization of the genomic regions for blast resistance and seedling vigor in rice (Takagi et al., 2013a); flowering QTL in cucumber (Lu et al., 2014) and fruit weight and locule number loci in tomato (Illa-Berenguer et al., 2015). In the case of legumes, this approach has also been found successful in the localization of QTLs/candidate genes for 100 seed weight in chickpea (Das et al., 2015). This study reported the identification of coding SNP in potential seed weight-governing candidate gene *CSN8*. Similarly, based on precise phenotyping of the cross ICC 4958 × ICC 1882, and sequencing of extreme bulks along with resistant parent were used to define candidate genes for 100-seed weight and root trait ratio (see Varshney, 2016). This approach has also found promising for identification of candidate genomic regions for late leaf spot and rust resistance in groundnut (see Varshney, 2016). In addition, this approach was tested in pigeonpea for localization of genomic regions for days to flowering and obcordate leaf shape (see Varshney, 2016).

The second approach called “MutMap” is a simple and robust NGS-based approach which was proposed in rice for the identification of candidate genes from promising EMS-induced mutants (Abe et al., 2012). The main advantage of this approach is that the mapping population developed for MutMap experiment requires crossing of selected mutant plant with the wild type, which minimizes the background noise. Thereafter, sequencing of extreme pool samples from segregating mapping populations along with a wild type parent are utilized for calculation of the genome-wide SNP index. Identification of SNP-index through specialized pipelines is useful for identification of candidate genes. Few more variants of MutMap approach are MutMap+ (Fekih et al., 2013; mapping without development of mapping population) and MutMap-Gap (Takagi et al., 2013b; useful approach for identification of candidate genes in the gap region, which was not sequenced through genome sequencing). Recently, MutMap approach has been found useful in identifying candidate gene for salinity tolerance leading to development of salt tolerance line through MABC (Takagi et al., 2015). In the context of legumes, this approach is being utilized for identification of candidate genes for leaf and plant type mutants in chickpea. Identified promising mutants in the genetic background of ICC 4958 are selected and crossed with wild-type parent. The developed M_2_ populations are being phenotyped for the targeted traits. Based on the phenotypic datasets extreme pools will be constructed for sequencing and candidate gene identification. In addition to chickpea, EMS induced mapping populations are being developed in the case of pigeonpea and groundnut.

The third approach namely “Seq-BSA” is NGS-based simple and robust approach for identification of candidate SNPs in the targeted genomic regions. This approach works on the calculation of genome-wide SNP-index of both the extreme bulks using high trait parent as reference parent assembly using QTL-seq pipeline (Takagi et al., 2013a). Identified SNPs that were monomorphic for high trait parent and high trait bulk will show SNP index of ‘0’ due to the presence of a similar genomic region of a particular locus. However, identification of the SNP index value of ‘1’ in low trait bulk, with the same genomic positions might be the putative SNPs linked to the target traits. This approach has been successfully utilized for the identification of putative SNPs associated with FW and SMD in pigeonpea (Singh V.K. et al., 2015).

The fourth approach namely “Indel-Seq” also has emerged as the promising trait mapping approach which is largely based on insertions and deletions. To date, the proposed approaches for identification of genomic regions are based on identification of SNPs and thereafter utilization of different statistical approaches for a declaration of candidate genomic regions/genes. However, the presence of insertions and deletions in the candidate genomic region has been largely ignored and not targeted for trait mapping in any of the approaches. This approach has larger practical utility with the fact that most of the cloned genes in rice and other crop possess Indels in the reported candidate genes.

In case of fifth approach namely “Bulked segregant RNA-Seq (BSR-Seq),” the strength of RNA-seq approach was combined with BSA and new genetic mapping approach to identify candidate genes for the target trait. This strategy has been successfully applied for the identification of *glossy3* genes of maize (Liu et al., 2012). Similar to this, RNA-seq of extreme pooled samples at high coverage were used to localize the candidate gene for grain protein content (GPC) gene *GPC-B1* in wheat (Trick et al., 2012). This approach has been found useful for the crops, which is having higher genome size (e.g., wheat 17 Gb and maize 2.3 Gb). This approach has more advantages in terms of cost saving as WGRS at higher coverage will be more costly than RNA-seq based experiments. Based on the merits of RNA-Seq, we are optimistic that this approach will be useful on the legumes with higher genome size (e.g., groundnut 2.5 Gb).

### Trait Mapping for Epigenetic Factors

Epigenetic markers associated with heritable epi-alleles for a particular trait can be deployed in crop breeding i.e., epigenomics-assisted breeding (EAB) (**Figure 1**). Whole genome bisulfite sequencing (WGBS) and chromatin immuno precipitation-sequencing (Chip-Seq) are the two important approaches for mapping epigenetic factors. In case of WGBS, the NGS based technologies enable us to find genome-wide 5-methylcytocine, in rapid and precise manner. Methylation of DNA cytosine plays a significant role in many cellular processes, including expression of the gene. DNA methylations have been playing an important role in the understanding of the molecular mechanism of heterosis. NGS-based WGBS approach has been found promising for understanding the molecular mechanism of heterosis in *Arabidopsis*, rice, and maize (He et al., 2010, 2013; Shen et al., 2012). Additionally WGBS approach has been successfully used to understand the segregation pattern of methylation in F_2_ generation (Schmitz et al., 2013). This approach is being utilized for understanding the molecular mechanism of heterosis in pigeonpea along with profiling of siRNA and transcriptome of parental lines and thereof derived hybrids.

In addition to WGBS, remodeling of chromatin through histone modification plays a significant role in the expression of many important genes. Specific histone modification has been found associated with expression of genes, plays a significant role in heterosis. In this context NGS-based, ChIP-seq approach was developed to identify the binding sites of DNA-associated proteins. This approach has been widely utilized in *Arabidopsis*, rice, and maize to find an association with heterosis (He et al., 2010, 2013; Shen et al., 2012). Along with WGBS, CHIP-seq approach is also being used in the case of pigeonpea hybrids and parental lines for understanding the molecular mechanism of heterosis.

## Transcriptomics Approaches for Gene Discovery and Marker Development

Identification of genes and pathways that are responsible for tolerance to various abiotic and biotic stresses is crucial to enhance productivity of legumes (**Figure 1**). Transcriptome sequencing is a better alternative approach to genome sequencing for targeted expressed gene sequencing. Furthermore, global gene expression analysis provides insights into gene function and molecular basis of various cellular components and transcriptional programs. Therefore, in legumes, to begin with, efforts have been focused on the development of cDNA libraries, generation of expressed sequence tags (EST), gene expression analysis, and the *in silico* mining of functional information from EST data sets even before genome sequences became available. The transcriptome sequencing has been applied for various other functional genomics approaches such as gene expression profiling, genome annotation and discovery of non-coding RNA, etc. (Morozova and Marra, 2008).

Extensive efforts have been made initially in legume transcriptomics and an abundance of ESTs from a range of tissues, including from plants challenged by different stresses have been generated in soybean (1.5 million ESTs, Vodkin et al., 2004), Medicago (*Medicago truncatula*; 280,000, Cheung et al., 2006) and Lotus (*Lotus japonicas*; 242,000, Asamizu et al., 2004). Initially, Sanger sequencing based ESTs were generated in majority of cases. For example, in the case of chickpea, cDNA libraries resulting in 20,162 ESTs have been generated from plants under drought and salinity stress conditions (Varshney et al., 2009). In the case of pigeonpea, responsive ESTs (9,888) for FW and SMD were generated (Raju et al., 2010). In addition, EST libraries have also been constructed using the suppression subtractive hybridization (SSH) technique, and utilizing this approach in chickpea, 477 drought-responsive ESTs were generated from root tissues (Buhariwalla et al., 2005). Further, Deokar et al. (2011) also generated 3,062 unigenes from SSH libraries of root and shoot tissues from drought tolerant- and sensitive- genotypes in chickpea. Similarly, in pigeonpea, 182 unique ESTs were generated from drought-stressed and unstressed pigeonpea seedlings using SSH (Qiao et al., 2012). However, major disadvantage of this method is that it is technically demanding and labor intensive.

In the recent years, several sequencing platforms available at low cost have already been demonstrated for use in generation of a huge set of transcript reads from a range of developing and stress–responsive tissues in different crop legumes. For instance, in chickpea, an improved transcriptome assembly has been generated based on FLX/454 sequencing together with Sanger ESTs comprised 103,215 Transcript Assembly Contigs (TACs) with an average contig length of 459 bp (Hiremath et al., 2011). In a different study, using the Illumina sequencing platform, another 53,409 contigs representing ∼28 Mb of unique transcriptome sequence were assembled (Garg et al., 2011b). The same group, by using both FLX/454 and Illumina sequencing technologies, defined another set of 34,760 contigs representing ∼4.8% (35.5 Mb) of the chickpea genome (Garg et al., 2011a). Furthermore, by analyzing sequencing data from three different platforms (Sanger, FLX/454, and Illumina), hybrid comprehensive assemblies have been generated in the case of pigeonpea (Kudapa et al., 2012) and chickpea (Kudapa et al., 2014). Several versions of transcriptome assemblies have been developed, in fact, for many different legume crops (see Varshney et al., 2015a). Furthermore, the National Center for Genome Resources (NCGR) in cooperation with the U.S. Department of Agriculture (USDA)-supported Legume Information System^2^ offers a comprehensive collection of transcriptome assemblies for several legumes.

Transcriptome assemblies generated using different sequencing technologies or in combination of two or more sequencing technologies provide valuable transcriptomic resources such as functional markers [EST-SSRs, SNPs, Intron Spanning regions (ISRs), etc.] for use in crop breeding programs. For example, a total of 1,682 and 4,099 SNPs were identified in soybean (Deschamps and Campbell, 2010) and common bean (Wu et al., 2014), respectively. In some studies, ISR markers (flanking intron junctions) were identified based on alignment to related genome sequences (Hiremath et al., 2011; Kudapa et al., 2012) and have found wide application in generating highly informative dense genetic maps with well distributed markers (Bordat et al., 2011). Furthermore, gene expression profiling data from the developed transcriptome assemblies would enable identification of candidate genes associated with different traits of interest including stress responsive genes. In common bean, a total of 441 salt responsive transcription factors (TF) were identified from a set 2,678 TFs classified under 59 TF families (Hiz et al., 2014) responsive. Information on the transcriptomic resources and candidate genes should provide insights into the molecular mechanisms of stress tolerance and ultimately help to develop improved stress tolerant legume varieties.

## Proteomics and Metabolomics

Advances in ‘omics’ technologies provide opportunities through which new datasets can be produced for crop plants. This is particularly important in crop research to identify candidate genes and pathways involved in important agronomic traits, especially through the integration of genomics and functional ‘omics’ data together with genetic and phenotypic information (Langridge and Fleury, 2011) (**Figure 1**). The above developments will ensure higher integration of ‘omics’ information with crop breeding leading to evolution from GAB to omics-assisted breeding (OAB) in coming years.

### Proteomics Approaches in Legumes

Proteomics involves research on cellular proteomes in which sets of protein species found in a biological unit such as cells/tissues/organs/organelle, at a particular developmental stage or external condition are analyzed (Jorrin et al., 2006). The increased range of proteome coverage and improvements in quantitative measurements have been vital for studying proteome composition, modulation, and modifications for development stages and stress–response mechanisms in plant system. In crops research, proteomics pipelines are increasingly being used, especially to study traits and stress response mechanisms, specific to crop systems. The proteomic information obtained, combined with the accurate identification of genetic determinants underlying a trait of interest together with improvements in the quality of genomic information, can be integrated into advanced breeding programs (Vanderschuren et al., 2013).

The important areas of plant proteomics include proteome mapping, comparison of protein profiles for different genotypes/biological units/stress factors (comparative proteomics), identification of posttranslational modifications (PTMs) and interaction networks through protein–protein interactions (PPI; Hu et al., 2015; Katam et al., 2015). Translational proteomics has great potential to be implemented in crop research for increasing agricultural production through the use of methodology and knowledge (Jorrin-Novo et al., 2015). Information obtained through orthoproteomics (different species) and comparative proteomics (different genotypes) approaches will have importance in advanced breeding programs through coordination and standardization of proteomic approaches and high quality crop proteomics databases (Vanderschuren et al., 2013).

Advances in mass spectrometry (MS) applications in terms of speed, accuracy, sensitivity, and software tools have been instrumental for high-throughput protein quantification. Quantitative proteomics platforms have emerged due to advances in MS technologies for high-throughput protein quantifications and involve gel-based or gel-free, ‘shot-gun’ and ‘label-based’ (isotopic/isobaric) or ‘label-free’ approaches (Abdallah et al., 2012; Hu et al., 2015). Gel-based proteomics involving two dimensional gel-electrophoresis (2D-GE) and difference-GE (2D-DIGE) for protein separation followed by MS analyses, have underpinned the understanding of proteomic changes during growth and development as well as stress response mechanisms in plants. Although 2D-GE has been the workhorse for protein expression analysis due to its high resolving power, 2D-DIGE, which involves pre-electrophoretic labeling of samples with fluorescent dyes, and allows separation of proteins on the same gel, is quantitatively more accurate. (Schulze and Usadel, 2010; Vadivel and Kumaran, 2015). Shotgun proteomics, is a ‘bottom–up, peptide-centric’ strategy, mostly involve coupled liquid chromatography tandem MS (LC–MS/MS) platforms, which is efficient for high-throughput analyses of cell or organelle proteome by providing an overview of the major proteins (Jorrin-Novo et al., 2015). Selected reaction monitoring (SRM) of peptides is a sensitive and specific targeted proteomic approach, to quantify the abundance of selected target proteins. It can also be an important method for biomarker validation of candidate proteins in dissecting molecular mechanisms underlying a particular trait for crops (Jacoby et al., 2013). PPI network analysis is an important aspect of proteomics by providing important information on the molecular mechanisms of signal transduction, protein complexes, stress responses as well as insight into developmental and physiological processes. The strategies involved include yeast-two hybrid (Y2H), affinity purification MS (AP-MS) and biomolecular fluorescence complementation (BiFC; Zhang et al., 2010).

Proteomics studies in legumes have been carried out mainly in the model systems, Medicago and Lotus (Watson et al., 2003; Larrainzar et al., 2007; Colditz, 2013; Lee et al., 2013; Dam et al., 2014; Ino et al., 2014) or soybean (Hossain et al., 2013; Hossain and Komatsu, 2014). Lesser but important proteomic information is available from the crop legumes such as protein differential expression to understand abiotic and biotic stress responses and proteome reference maps. Although there is limited information available currently, proteomics based datasets for the crop legumes will be enhanced with the increasing availability of legume genome sequences and transcriptome datasets (Dubey et al., 2011; Hiremath et al., 2011; Kalavacharla et al., 2011; Varshney et al., 2013c). Examples of proteome reference maps available in crop legumes include different subcellular membrane proteins from chickpea (Jaiswal et al., 2012), mature seed proteins and vegetative tissue proteins from pea (Schiltz et al., 2004; Bourgeois et al., 2009) and leaf proteins from groundnut (Katam et al., 2010).

In crop legumes, differential expression analyses and comparative proteomics approaches have provided insight to stress responses. Some examples include dehydration in chickpea (Bhushan et al., 2007; Pandey et al., 2008; Subba et al., 2013), early phases of cold stress in chickpea (Heidarvand and Maali-Amiri, 2013), drought stress in common bean (Zadraznik et al., 2013), fungal infection in pea (Barilli et al., 2012), drought stress in groundnut (Basha et al., 2007; Kottapalli et al., 2008), and salt stress in groundnut calli (Jain et al., 2006). For studying PTMs, in chickpea, a nucleus-specific phosphoproteome map was generated in developing seedlings (Kumar et al., 2014) and phosphoproteins were also analyzed in root tips of common bean subjected to osmotic stress (Yang et al., 2013).

### Metabolomics Approaches

Metabolomics approaches involve the identification and quantification of low molecular weight metabolites in an organism, at a particular developmental stage in a specific organ/tissue/cell and have been successfully implemented to investigate the molecular phenotypes of plants in response to abiotic stress (Arbona et al., 2013). High-throughput screening of metabolites are advantageous compared to targeted reverse genetic approaches for plant metabolic engineering because the former method provides a more comprehensive understanding of metabolic networks in connection with developmental stages of phenotypes and capable of screening out unwanted traits (Fernie and Schauer, 2009).

The two main metabolomics profiling strategies using MS and nuclear magnetic resonance (NMR) have been described in the literature. A wider coverage of the great number of metabolites in plants has been obtained through the combination of several analytical techniques that usually consist of a separation techniques coupled to MS (for detection; Arbona et al., 2013). The regularly applied methods are gas-chromatography–MS (GC–MS), GC–time of flight–MS (GC-TOF-MS) and LC–MS are regular methods. GC–MS allows the identification and quantification of large number of primary metabolites, GC–TOF–MS is noted for its fast scan times for better resolved peaks and sample throughput, while LC–MS allows a broader range of primary and secondary metabolites to be measured (Fernie and Schauer, 2009). Other techniques include flow injection analysis coupled to MS (FIA/MS) and Fourier Transform Infrared spectroscopy (FTIR; Arbona et al., 2013).

In legumes, most of the metabolic profiling analyses have been carried out in the model systems, Medicago and Lotus. A non-targeted, comparative metabolomic was used to study drought tolerance in Lotus genotypes in which conserved and unique metabolic responses were identified, with GC coupled to electron impact ionization (EI)–TOF–MS for metabolic profiling (Sanchez et al., 2012). A couple of other examples of metabolic profiling in Lotus include analysis of flavonoids (Suzuki et al., 2008) and long term salt stress response mechanisms (Sanchez et al., 2011). To study legume–rhizobia symbiosis in Medicago, which involves rapid metabolic changes in both partners, untargeted quantitative, LC–electrospray ionization (ESI)–TOF–MS was used for metabolic profiling followed by targeted LC–ESI–QTrap MS of selected candidates metabolites (Zhang et al., 2012). The study was useful in identifying that oxylipin pathway being involved in nodulation factor signaling in the early stages of symbiosis. In Medicago, some of the other recent examples include the study of new pathways and alternative mechanism for phenylpropanoid and isoflavonoid biosynthesis (Farag et al., 2008), development and symbiosis-dependent primary and secondary metabolism in roots (Schliemann et al., 2008), metabolites involved in symbiosis (Ye et al., 2013). Among the crop legumes, fewer metabolic profiling studies available and include chickpea-Fusarium interaction (Kumar et al., 2015), developing pea seed (Vigeolas et al., 2008), phosphorus stressed common bean (Hernandez et al., 2009).

The integration of metabolomics with transcriptomics datasets, high-throughput phenotyping and bioinformatics platforms, for profiling of large and genetically diverse populations, will enable the identification of novel metabolic QTLs and enhance the identification of candidate genes for trait of interest. Also metabolomics used as an additional tool with genomics-assisted selection strategies for crop-improvement, reduces the times spent for the discovery of new traits and allelic variations (Fernie and Schauer, 2009).

## Breeding Informatics Tools

Breeding informatics tools are pre-requisites for effective and efficient application in each and every step in any molecular breeding program, starting from planning for field experiments to taking breeding decision for selection of plants in making the crosses. The GAB experiments involve identification of suitable germplasm from large scale diversity experiments to develop mapping population and identification of quantitative trait loci through genotyping and phenotyping using either family based mapping approach or germplasm based approach (association mapping). Identification of marker-trait associations through detailed analysis of genotypic and phenotypic data and finally applying markers in molecular breeding programs depends on a sequential use of a number of decision support tools (Xu, 2010). In this context, Breeding Management System (BMS) of Integrated Breeding Platform (IBP)^3^ has been developed to help breeders to manage their day-to-day activities through all phases of their breeding programs. The BMS Workbench is useful from straightforward phenotyping to complex genotyping. It provides all the tools needed to conduct modern breeding in one comprehensive package including, project planning, germplasm management, germplasm evaluation, molecular analysis, data analysis, and breeding decision support (Varshney et al., 2015b).

## Molecular Breeding for Accelerated Legume Improvement

The conventional breeding approaches led to development of large number of improved cultivars over the time for legumes. However, these improved cultivars lags far behind in attempting to match the ever increasing human population and thus putting more pressure on the ongoing breeding programs across the world. Further, the limiting natural resources in terms of land and water in addition to climate change and uncertain rains have further made the ongoing breeding programs on the high pressure. Nevertheless, last decade has witnessed a speedy development in the area of NGS and high-throughput genotyping technologies. These developments led to development of trait-linked markers for several traits using first generation of markers (RFLP, RAPD, SSR) and mapping populations (F_2_, RIL, NIL, BIL, DH and germplasm sets). There are three GAB approaches namely MABC, marker-assisted recurrent selection (MARS), and GS which are currently been deployed for developing improved lines (**Figure 1**).

The MABC has been the most preferred and result oriented molecular breeding approach for improving existing popular genotypes for one or two traits and pyramiding of few genes/QTLs. This approach has become very popular and widely accepted among breeders for these reasons; optimum time utilization and resources, opportunity to perform selection at early growth stages for multiple genes/loci, ability to break linkage between desirable and non-desirable traits, and most importantly no phenotyping requirement except for final trait confirmation. In the case of chickpea, MABC has been successfully used for introgressing a “*QTL-hotspot*” harboring several QTLs controlling several drought tolerance related root traits in the elite chickpea variety JG 11 (drought tolerant variety; Varshney et al., 2013a). Introgression lines have shown improved performance under rainfed as well irrigated environment as compared to recipient parent. In another attempt in chickpea, this approach was used for introgression of resistance to FW and ascochyta blight (AB) into the elite chickpea cultivar C 214 (Varshney et al., 2013b). Further, now the efforts are underway to pyramid the resistance to FW and AB by crossing introgression lines. Similarly in the case of groundnut, diagnostic markers were used to develop an improved cultivar called ‘NemaTAM,’ which is the first RKN resistant groundnut variety and was released for cultivation in the USA (Simpson et al., 2003). In an another effort, high oleate trait was improved in the nematode resistant cultivar “Tifguard” leading to development of second cultivar “Tifguard High O/L” possessing resistance to nematode and high oleate trait (Chu et al., 2011). Most recently, three elite and popular cultivars (TAG 24, JL 24, and ICGV 91114) were improved for rust resistance while three elite cultivars were improved for oil quality (Varshney et al., 2014a; Janila et al., 2016). The above achievements have demonstrated significance of molecular breeding and many research organizations across the world are deploying molecular breeding in their crop improvement programs. Further availability of linked markers in future from trait mapping pipeline will provide more options to the breeders in accumulating favorable alleles for multiple traits in a single genetic background using MABC approach.

Another molecular breeding approach termed MARS promises improve to accumulate superior alleles for quantitative traits such as drought resistance, yield, etc., which are controlled by several QTLs, each with a small effect on the phenotype and the ideal genotype cannot be attained through MABC (Varshney et al., 2013c). In such situations, MARS is quite useful to target more number of minor as well as major QTLs (Ribaut and Ragot, 2007). MARS approach requires estimation of marker effects for traits of interest and then favorable alleles linked to these traits are traced in two or three recombination cycles to combine favorable alleles (Bernardo and Charcosset, 2006). Multiallelic MARS which involves creating a series of bi-allelic lines and finally assembling them in the final inbred line has also been suggested for traits where the number of target loci is small (Ribaut and Ragot, 2007). In the case of chickpea, in order to pyramid favorable alleles for drought tolerance, two MARS crosses were initiated (JG 11 × ICCV 04112 and JG 130 × ICCV 05107). Based on QTL analysis on genotyping data on F_3_ lines and phenotyping data on F_5_ lines, superior lines were selected using OptiMAS ver. 1.0 (Valente et al., 2013). Although, MARS have been proven successful in private breeding programs for enhancing genetic gain, it could not achieve similar level of success in chickpea.

Another emerging molecular breeding approach called GS holds great promise in achieving higher genetic gains in lesser time for complex traits. Unlike MABC and MARS, the GS approach does not require population development and identification of linked markers prior to its deployment and has potential to enhance genetic gains for even complex traits such as yield under drought stress. This approach deploys evenly distributed genetic markers across the genome to predict GEBV using multiple methods with varying degrees of complexity, computational efficiency, and predictive accuracy (Meuwissen et al., 2001). For achieving higher genetic gains, GS showed its potential in accumulating 1000s of favorable alleles leading to development of resilient crop varieties with high yield potential under unfavorable conditions. Most importantly, GS can reduce breeding cost by 22.4% and breeding duration which is a remarkable achievement (König et al., 2009). ICRISAT has taken initiatives by constituting TPs in chickpea and groundnut with 320 and 314 elite breeding lines, respectively. These populations have been genotyped with DArT arrays (15,360 features) and are also being used currently to generate multi-season and multi-location phenotyping data on important traits. In parallel, the effort is also underway to genotype these populations with 60 K crop specific SNP array. The high-throughput genotyping and phenotyping data on these sets will be used for development and train appropriate GS models to initiate GS breeding in chickpea and groundnut. Similarly in case of pigeonpea wherein the emphasis is more on development of hybrids, a set of cytoplasmic male sterility (CMS) lines and restorer lines have been included in the TP. In this context, a total of 550 test cross F_1_s have been developed through hybridization using 55 restorer lines and 10 CMS lines for phenotyping at multiple locations. Based on the genotypic datasets of parental lines and phenotypic datasets of test cross F_1_s, the GS models will be defined for use in GS breeding to develop improved parental lines and new hybrids in pigeonpea.

## Conclusion

The demand-supply gap for the legumes is perpetually increasing widening day-by-day which will lead to a huge shortfall in the supply to the ever increasing global population in coming years. The only option is to maximize the efforts toward developing improved high yielding cultivars possessing resistance/tolerance to the major stresses especially in context of climate change. The cost-effective sequencing technologies have introduced a new era in genomics and breeding by pinpointing the genes responsible for distinct phenotypes leading to selection of plants based on genotyping information. The knowledge generated through all the “omics” studies need to be integrated in breeding so that breeders can move toward “knowledge-based breeding” from “chance breeding.” In recent years, several emerging genomic technologies have been developed to foster trait mapping, gene discovery, and trait improvement programs in several crop plants. Some of these technologies have been already deployed in the three legume crops as discussed in this review. In addition, important information generated through transcriptomics, proteomics, metabolomics, and epigenomics have greatly benefitted the scientific community in developing better understanding of the traits and crops leading to development of effective strategy for achieving higher genetic gains in less time. It is obvious that the current and the upcoming technologies will further assist legume improvement programs in a more cost-effective, user-friendly, and less time consuming manner.

## Author Contributions

MP planned the MS content, coordinated with other coauthors, contributed in drafting special sections, brought flow between different sections, and finalizing the manuscript. MR, VS, AbiR, HK, MT, AC, and AbhR contributed specific sections of this manuscript. MP, MR, and VS worked on this MS in several rounds to bring in proper shape. RV was involved in planning the content and finalizing the manuscript.

## Conflict of Interest Statement

The authors declare that the research was conducted in the absence of any commercial or financial relationships that could be construed as a potential conflict of interest.

## Footnotes

1 http://www.generationcp.org/gcp-research/crops/legumes

2 http://www.comparative-legumes.org

3 https://www.integratedbreeding.net/
